# A Large‐Scale Serological Survey in Pets From October 2020 Through June 2021 in France Shows Significantly Higher Exposure to SARS‐CoV‐2 in Cats Compared to Dogs

**DOI:** 10.1111/zph.13198

**Published:** 2024-12-08

**Authors:** Matthieu Fritz, Eric Elguero, Pierre Becquart, Daphné De Riols de Fonclare, Déborah Garcia, Stephanie Beurlet, Solène Denolly, Bertrand Boson, Serge G. Rosolen, François‐Loïc Cosset, Alexandra Briend‐Marchal, Vincent Legros, Eric M. Leroy

**Affiliations:** ^1^ Maladies Infectieuses et Vecteurs, Ecologie, Génétique, Evolution et Contrôle (MIVEGEC) Univ. Montpellier, IRD, CNRS Montpellier France; ^2^ Laboratoire de Biologie vétérinaire VEBIO Arcueil France; ^3^ CIRI – Centre International de Recherche en Infectiologie, Team EVIR Univ Lyon, Université Claude Bernard Lyon 1, Inserm, U1111, CNRS, UMR5308, ENS Lyon Lyon France; ^4^ Sorbonne Université, INSERM, CNRS Institut de la Vision Paris France; ^5^ Clinique vétérinaire Voltaire Asnières France; ^6^ Université de Lyon, VetAgro Sup, Campus vétérinaire de Lyon Marcy‐l'Etoile, Lyon France

**Keywords:** microsphere immunoassay, neutralisation assay, one health, pets, SARS‐CoV‐2, serology

## Abstract

**Introduction:**

Severe acute respiratory syndrome coronavirus 2 (SARS‐CoV‐2) has the potential to infect various animals, including domestic pets like dogs and cats. Many studies have documented infection in companion animals by molecular and serological methods. However, only a few have compared seroprevalence in cats and dogs from the general population, and these studies were limited by small sample sizes and collections over short periods. Our aim was to obtain a more accurate evaluation of seroprevalence in companion animals in France and to determine whether cats and dogs differ in their exposure to SARS‐CoV‐2.

**Methods:**

We conducted an extensive serological survey of SARS‐CoV‐2, collecting blood samples from 2036 cats and 3577 dogs during routine veterinary medical examinations across different regions of metropolitan France from October 2020 to June 2021. This period encompassed the peaks and onset of two waves, as well as the emergence of the first variants. A microsphere immunoassay targeting the receptor‐binding domain and trimeric spike protein was used to detect anti‐SARS‐CoV‐2 antibodies. A subset of 308 seropositive samples was tested for the presence of neutralising antibodies.

**Results:**

We determined an overall seroprevalence of anti‐SARS‐CoV‐2 antibodies of 7.1% (95% confidence interval [CI]: 6.4%–7.8%) among the sampled pets. Cats exhibited a significantly higher seroprevalence (9.3%; 95% CI: 8.1%–10.1%) compared to dogs (5.9%; 95% CI: 5.2%–6.8%). Among the subset of seropositive samples, 81 (26.3%; 95% CI: 21.5%–31.6%) displayed neutralizing antibodies. Furthermore, seroprevalence in both species was lower in older animals and was not associated with sex. Finally, unlike cats, seroprevalence in dogs was found to be correlated with the date of sampling.

**Conclusions:**

The large sample size enhances the reliability and statistical robustness of our estimates regarding pet exposure to SARS‐CoV‐2. This study on SARS‐CoV‐2 reaffirms the crucial importance of adopting a One Health approach incorporating domestic animals when managing an epidemic caused by a zoonotic virus.

**Summary**

- **Heightened Awareness:** Our study highlights the significant prevalence of SARS‐CoV‐2 exposition in pets, urging pet owners to remain vigilant and take appropriate precautions to protect both human and animal health.
- **Public Health Vigilance:** With the potential for human‐to‐pet transmission, our findings emphasise the need for robust surveillance and preventive measures among companion animals and mitigate the risk of viral mutation due to cross‐species transmission.
- **One Health Approach:** Our research underscores the importance of a collaborative One Health approach to effectively combat zoonotic diseases like COVID‐19.

## 1 Introduction

Two months after the onset of SARS‐CoV‐2 circulation in humans, two dogs in Hong Kong were reported to have naturally acquired the virus (Sit et al. 2020). Since then, many studies have reported viral RNA and SARS‐CoV‐2 antibodies in dogs and cats—mostly belonging to COVID‐19‐infected owners (Barrs et al. 2020; Goryoka et al. 2021; Hamer et al. 2021; Jairak et al. 2021; Krafft et al. 2021). Furthermore, several studies demonstrated that the risk of pets testing seropositive was higher in COVID‐19+ households than for pets from households of unknown status (Barroso et al. 2022; Colitti et al. 2021; Fritz et al. 2021a; Fritz et al. 2021b; Patterson et al. 2020; Stevanovic et al. 2021).

Definitive examples of pet‐to‐human transmission are scarce. Studies from Thailand reported a suspected case of SARS‐CoV‐2 transmission from a cat to a human (Piewbang et al. 2022; Sila et al. 2022), and dog‐to‐human transmission has yet to be described. However, given that 200 million cats and dogs live in close proximity to humans in Europe (The European Pet Food Industry 2020), there is ample opportunity for such transmission, and the potential risks need to be carefully considered.

Several population‐based serological studies have reported SARS‐CoV‐2 antibodies in dogs and cats. In dogs, estimates of seroprevalence have ranged from 0% to 14.5% (Barroso et al. 2022; Barroso‐Arévalo et al. 2021a; Barroso‐Arévalo et al. 2021b; Barua et al. 2021; Dileepan et al. 2021; Laidoudi et al. 2021; Pomorska‐Mól et al. 2021; Smith et al. 2021; Stevanovic et al. 2021; Stevanovic et al. 2020; Udom et al. 2021; Zhao et al. 2021). In cats, estimates have ranged from 0% to 21.7% (Barroso et al. 2022; Barua et al. 2021; Dileepan et al. 2021; Michelitsch et al. 2020; Michelitsch et al. 2021; Schulz et al. 2021; Udom et al. 2021; Zhang et al. 2020). For both species, seroprevalence was highly dependent on the period of sampling (first wave, second wave, etc.), the assay used (ELISA, seroneutralisation, etc.) and the country of sampling (China, Croatia, Germany, Italy, the Netherlands, Poland, Portugal, Spain, Thailand, United Kingdom, USA). Among these studies, five directly compared cats and dogs. There is some experimental and epidemiological evidence suggesting that cats are more susceptible to infection than dogs (Barroso et al. 2022; Bosco‐Lauth et al. 2020; Colitti et al. 2021; Dileepan et al. 2021; Shi et al. 2020). However, significant species differences have not always been observed in population‐based studies (Barroso‐Arévalo et al. 2021a; Barroso‐Arévalo et al. 2021b; Pomorska‐Mól et al. 2021; Stevanovic et al. 2020). This is perhaps because of significant study limitations—a low number of enrolled animals, a short sampling period, etc.—that have curtailed robust estimates of infection frequency in pets with enough statistical power to recognise differences in COVID‐19 epidemiology.

Understanding the prevalence of anti‐SARS‐CoV‐2 antibodies in pets is still relevant for assessing the risk of zoonotic transmission and implementing effective public health measures. The primary objective of this study is to assess the seroprevalence of anti‐SARS‐CoV‐2 antibodies within a substantial cohort of French domestic dogs and cats. Additionally, we aim to elucidate any potential factors associated with seropositivity in this population.

## 2 Materials and Methods

### 2.1 Sampling and Blood Collection

The cross‐sectional study was developed in collaboration with a nationwide network of veterinary clinics working with VEBIO, a veterinary diagnostic laboratory performing all categories of medical analyses, including infectious diseases, haematology, endocrinology and oncology (see more details in https://www.vebio.fr/). The sampling process involved the collection of blood samples from dogs and cats during routine healthcare visits or diagnostic procedures at participating veterinary clinics. No inclusion or exclusion criteria were applied during sample collection, except that samples came only from veterinary clinics working with VEBIO. VEBIO notified the veterinary clinics that following requested biomedical analyses, the remaining serum could be used in a SARS‐CoV‐2 research project. No specific request for samples was addressed to the vets. Thus, the SARS‐CoV‐2 analysis is based on samples collected during the regular activities of the vets.

**FIGURE 1:**
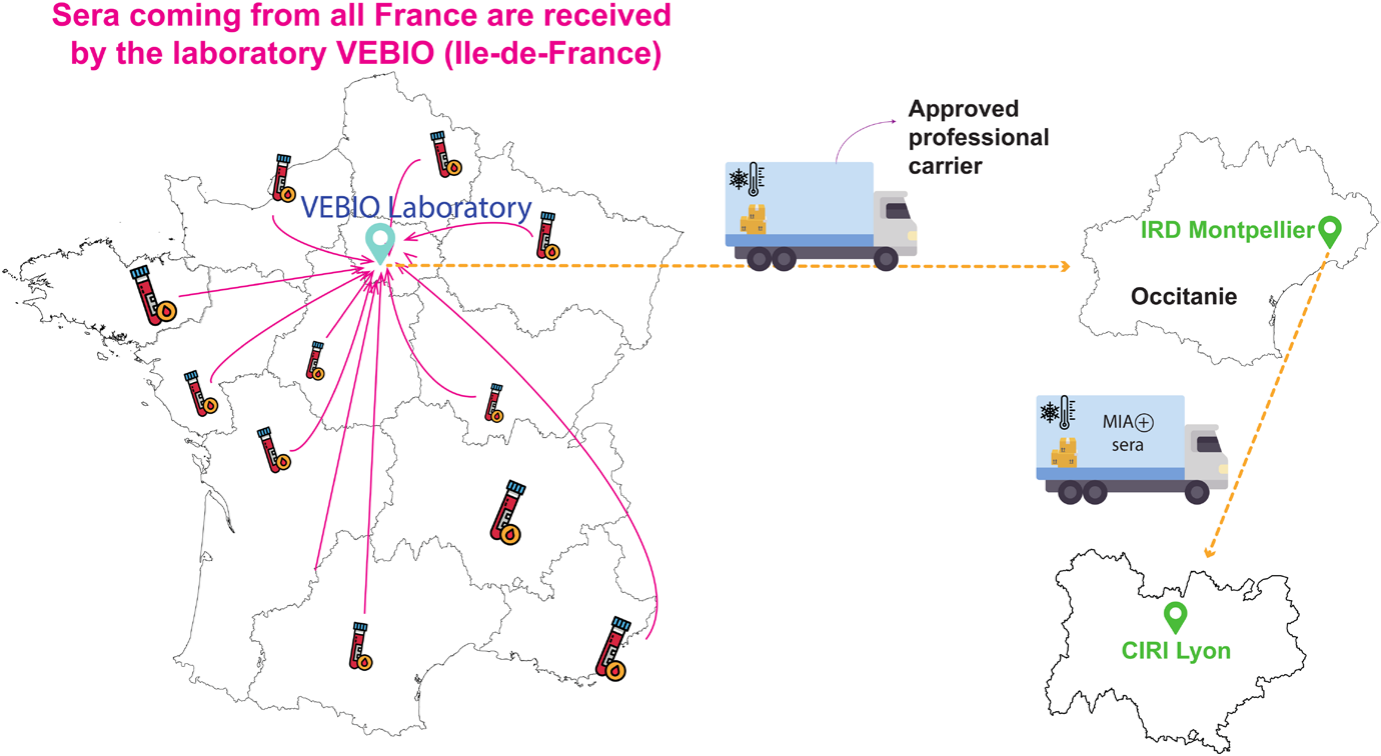
Logistics of sample collection and distribution. Sera collected during routine healthcare visits by veterinarians throughout France were first sent to VEBIO in Ile‐de‐France. Aliquots of the samples were then made and sent to the IRD in Montpellier (Hérault) via an approved carrier.

Blood samples were collected in dry/EDTA tubes from 5613 pets (2036 cats and 3577 dogs) during routine healthcare visits or for diagnostic purposes at veterinary clinics from October 2020 through June 2021 (Table 1). Samples were collected from 12 regions of metropolitan France (excluding Corsica). Almost half of the samples came from Ile‐de‐France, the region including Paris, reflecting population density and proximity to the veterinary diagnostic laboratory (VEBIO). After centrifugation, the serum/plasma was kept at +4°C until sent to VEBIO. Rapid and safe shipping practices were used to avoid contamination and ensure samples reached VEBIO within 48 h. At the VEBIO facility, an aliquot was taken from the sample to perform the requested biomedical analyses. Another aliquot was then stored at +4°C until sent to the MIVEGEC lab, Montpellier, where serological analyses were performed. Safe shipping practices with an approved professional carrier were also used for shipment to the MIVEGEC lab. Finally, the samples were stored at the MIVEGEC lab at −20°C until testing (Figure 1). For shipping to the CIRI lab, SARS‐CoV‐2‐positive samples detected by MIA were transported by an approved professional carrier at −20°C to ensure optimal safety conditions. Data (age, sex, clinical history and region localisation recorded by the veterinarian, when available) from dogs and cats were provided anonymised by VEBIO to the MIVEGEC lab.

**TABLE 1:** Numbers of samples collected by month and by species.

	October 2020	November 2020	December 2020	January 2021	February 2021	March 2021	April 2021	May 2021	June 2021	Total
Cats	49	256	275	291	225	296	305	166	173	2036
Dogs	84	428	543	475	403	474	597	282	291	3577
All	133	684	818	766	628	770	902	448	464	5613

### 2.2 Ethics

In accordance with the law governing the use of live animals for scientific purposes in France, effective as of January 14, 2022, ethical approval was neither sought nor necessary, as all pet samples were collected by veterinarians during routine healthcare visits. In the context of this study, no informed consent from pet owners was required for the use of the samples. All applicable international and national guidelines for the care of pets were followed.

### 2.3 Microsphere Immunoassay (MIA)

Dog and cat serum samples were tested using a multiplex microsphere immunoassay (MIA). Ten micrograms of two recombinant SARS‐CoV‐2 antigens, receptor‐binding domain (RBD) and trimeric spike (tri‐S), both derived from the Wuhan‐Hu‐1 strain (The Native Antigen Company, Kidlington United Kingdom), were used to capture specific serum antibodies. Distinct MagPlex microsphere sets (Luminex Corp, Austin, TX, USA) were respectively coupled to viral antigens using the amine coupling kit (Bio‐Rad Laboratories, Marnes‐la‐Coquette, France) according to the manufacturer's instructions. Microsphere mixtures were successively incubated with serum samples (1:400), biotinylated protein A and biotinylated protein G (4 μg/mL each) (Thermo Fisher Scientific, Illkirch, France), and streptavidin‐R‐phycoerythrin (4 μg/mL) (Life technologies, Illkirch, France) on an orbital shaker and protected from light. Measurements were performed using a Luminex 200 instrument (Luminex Corp, Austin, TX, USA), and at least 100 events were read for each bead set. Binding events were displayed as median fluorescence intensities (MFIs). MIA was first validated using sera from two COVID‐19 PCR+ humans, kindly provided by Meriadeg Ar Gouilh. Subsequently, validation was conducted using sera from SARS‐CoV‐2 PCR+ cats and dogs obtained from previous studies (Ferasin et al. 2021; Fritz et al. 2021a; Fritz et al. 2021b), which were provided by several veterinarians. Specific seropositivity cut‐off values for each antigen were set at three standard deviations above the mean MFI of pre‐pandemic serum from 53 dogs and 30 cats sampled before 2019. These samples were stored in biobanks at the IRD and VetAgro Sup. MIA specificity was set for each antigen at 96.2% for dogs and 100% for cats based on the pre‐pandemic populations.

Because of the excellent specificity observed for both antigens and to account for any isotypic variability, an animal was deemed positive for SARS‐CoV‐2 antibodies following a positive result in at least one of the two tests.

### 2.4 Neutralisation Activity Measurement

For logistical reasons, a subset of 308 MIA‐positive serum samples was randomly selected, without applying specific criteria, for testing using an MLV‐based pseudoparticle carrying a GFP reporter pseudotyped with the SARS‐CoV‐2 spike protein (Wuhan‐Hu‐1 strain) (SARS‐CoV‐2 pp). This was done to evaluate neutralising antibody activity in sera from cats and dogs, following a neutralisation procedure previously outlined by (Legros et al. 2021). Briefly, for neutralisation assays, a sample of ~1 × 10^3^ pseudoparticles was incubated with a 100‐fold dilution of sera or control antibodies for 1 h at 37 °C before infection of Vero‐E6R cells. At 72 h post transduction, the percentage of GFP‐positive cells was determined by flow cytometry (at least 10,000 events recorded). The level of infectivity is expressed as the percentage of GFP‐positive cells and compared to cells infected with SARS‐CoV‐2 pp. incubated without serum. As a control, the same procedure was done using RD114 pseudoparticles to identify sera with aspecific neutralisation. Sera exhibiting more than 30% SARS‐CoV‐2 pp. neutralisation were considered positive. Pre‐pandemic serum from France was used as a negative control, and an anti‐SARS‐CoV‐2 RBD (Sinobiological) antibody was used as a positive control.

### 2.5 Statistical Analyses

Seroprevalence was described by percentages with 95% exact binomial confidence intervals (CIs). Associations between SARS‐CoV‐2 exposure status (positive or negative) and the covariate region, age, sex and time of sampling were assessed using binomial (logistic) generalised linear models. For all statistical analyses, odds ratios (ORs) with 95% CI were calculated based on logistic regression analysis to quantify the strength of these associations. *p*‐values were computed by the likelihood ratio test, and a threshold of ≤ 0.05 was considered statistically significant. All analyses were performed using R software (version 4.0.3)(R Development Core Team, 2018).

#### 2.5.1 For Seroprevalence in Cats and Dogs

We analysed the differences in SARS‐CoV‐2 prevalence between cats and dogs, taking into account geographic regions. To do this, we extracted unique regions from the available data and fitted binomial logistic regression models for each region. The region was defined by where the animal lived at the time of sampling. We used species (dog or cat) as the main explanatory variable. A global model was fitted to evaluate the association between species and SARS‐CoV‐2 infection across all regions.

#### 2.5.2 For Seroprevalence by Age or Sex

We investigated the association between age or sex and the probability of SARS‐CoV‐2 exposure in cats and dogs. Records with missing data on age or sex were excluded. Separate logistic regression models were constructed for each species (cats and dogs), with age or sex serving as predictor variables. We performed similar analyses for the combined dataset of cats and dogs. Additionally, logistic regression models were fitted for cats, dogs and the combined dataset with sex and age as predictor variables.

#### 2.5.3 For Seroprevalence Over Time

To investigate the impact of time of sampling on the likelihood of SARS‐CoV‐2 exposure among cats and dogs, we filtered the dataset to include only records with known age greater than or equal to one year. By limiting the analysis to animals at least one year old, we excluded younger animals born after the start of the pandemic, thereby ensuring that all animals had potentially experienced the same degree of exposure to the virus. This approach allowed for a more accurate comparison of seroprevalence at different times of sampling in the study. Subsequently, logistic regression models were fitted separately for cats and dogs, with time of sampling (measured as the number of days since 1 January 2020) and age as predictor variables. A null model was also fitted to assess the significance of adding time as a predictor.

## 3 Results

### 3.1 Global Seroprevalence

For the sera samples, 401 (7.1%; 95% CI[6.5%–7.8%]) showed a positive result against RBD, tri‐S, or both (Supplementary Table S1). We next determined the presence of antibodies with neutralising activity among 308 randomly selected positive sera (Materials and Methods). Seroneutralising activity was detected in 81 (26.3%; 95% CI [21.5%–31.6%]) of the 308 pet sera samples. Among these positive samples, 39 (48%) were positive for both RBD and tri‐S, 39 (48%) were positive only for tri‐s and 3 (4%) were positive only for RBD. Prior investigations have demonstrated that certain pets may exhibit a lack of production of neutralising antibodies (Ferasin et al. 2021; Krafft et al. 2021). Consequently, only the seroprevalence data obtained from MIA assays were considered for further analysis in this study.

### 3.2 Seroprevalence in Cats and Dogs

**FIGURE 2:**
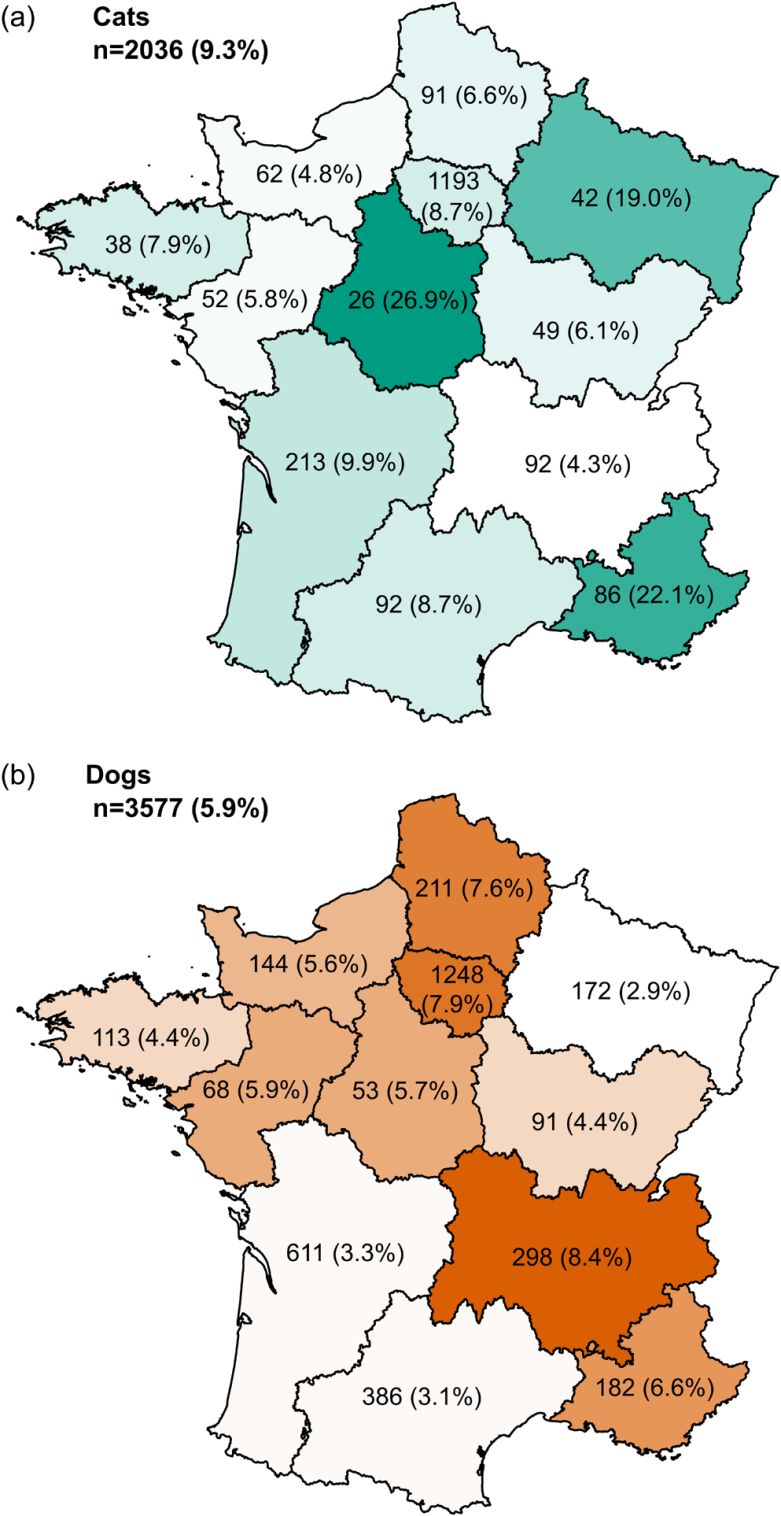
(a) Map of France showing the number of SARS‐CoV‐2‐positive cat sera per region. The total number of sera samples collected per region is indicated. Seroprevalence in each region is indicated as a percentage. Regions are shaded in green according to seroprevalence. The total number of sera samples and global seroprevalence for France is in the top left corner. (b) Map of France showing the number of SARS‐CoV‐2‐positive dog sera per region. The total number of sera samples collected per region is indicated. Seroprevalence in each region is indicated as a percentage. Regions are shaded in orange according to seroprevalence. The total number of sera samples and global seroprevalence for France is in the top left corner.

We observed that a significantly greater proportion of cats were positive (189/2036, 9.3%) than dogs (212/3577, 5.9%); OR = 1.62, 95% CI [1.32–1.99], *p*‐value = 3.8e‐06 (Figure 2 and Table 2). In addition, sera from MIA‐positive cats were more likely to show neutralising activity (49/144, 34%) than dogs (32/164, 19.5%); OR = 2.12 (95% CI [1.27–3.57], *p*‐value = 0.0039). Species differences were not always significant within each region. However, when differences were significant, it was always the case that cats were more likely to be positive than dogs (Table 2).

**TABLE 2:** Seroprevalence of IgG SARS‐CoV‐2 antibodies detected in blood samples from cats and dogs collected in different French regions from October 2020 through June 2021.

Region	Cats	Cats seroprevalence (95% CI)		Dogs	Dogs seroprevalence (95% CI)		OR (95% CI)	*p*‐value
Auvergne‐Rhône‐Alpes	4/92	4.3%	(1.2–10.8)	25/298	8.4%	(5.5–12.1)	0.50 (0.17–1.47)	1,72E‐01
Bourgogne‐Franche‐Comté	3/49	6.1%	(1.3–16.9)	4/91	4.4%	(1.2–10.9)	1.42 (0.30–6.61)	6,59E‐01
Bretagne	3/38	7.9%	(1.7–21.4)	5/113	4.4%	(1.5–10.0)	1.85 (0.42–8.14)	4,28E‐01
Centre‐Val de Loire	7/26	26.9%	(11.6–47.8)	3/53	5.7%	(1.2–15.7)	6.14 (1.44–26.23)	**9,82E‐03**
Grand Est	8/42	19.0%	(8.6–34.1)	5/172	2.9%	(1.0–6.7)	7.86 (2.42–25.49)	**5,66E‐04**
Hauts‐de‐France	6/91	6.6%	(2.5–13.8)	16/211	7.6%	(4.4–12.0)	0.86 (0.33–2.27)	7,59E‐01
Île‐de‐France	104/1193	8.7%	(7.2–10.5)	98/1248	7.9%	(6.4–9.5)	1.12 (0.84–1.49)	4,38E‐01
Normandie	3/62	4.8%	(1.0–13.5)	8/144	5.6%	(2.4–10.7)	0.86 (0.22–3.37)	8,32E‐01
Nouvelle‐Aquitaine	21/213	9.9%	(6.2–14.7)	20/611	3.3%	(2.0–5.0)	3.23 (1.72–6.09)	**3,66E‐04**
Occitanie	8/92	8.7%	(3.8–16.4)	12/386	3.1%	(1.6–5.4)	2.97 (1.18–7.49)	**2,81E‐02**
Provence‐Alpes‐Côte d'Azur	19/86	22.1%	(13.9–32.3)	12/182	6.6%	(3.5–11.2)	4.02 (1.85–8.73)	**3,61E‐04**
Pays de la Loire	3/52	5.8%	(1.2–15.9)	4/68	5.9%	(1.6–14.4)	0.98 (0.21–4.58)	9,79E‐01
Total	189/2036	9.3%	(8.1–10.6)	212/3577	5.9%	(5.2–6.8)	1.62 (1.32–1.99)	3.80E‐06

### 3.3 Seroprevalence by Age

Age was reported for 1657 cats (range: 0.2–22 yr) and 2781 dogs (range: 0.1–18.5 yr). Using a binomial model with age entered as a continuous variable, we observed a significant decrease in seroprevalence with age in cats (OR for a one‐year increase in age = 0.91, 95% CI [0.88–0.94], *p*‐value = 3.7e‐08) and dogs (OR = 0.95, 95% CI [0.92–0.99], *p*‐value = 0.016).

### 3.4 Seroprevalence by Sex

Sex was reported for 5203 pets (1836 cats and 3367 dogs). We found no significant sex differences in seropositivity rates, either for all animals (females: 6.9%; 163/2361; males 7.5%; 212/2842; *p* = 0.24) or among cats (females 9.4%; 78/827, males 9.8%; 99/1009, *p* = 0.68) and dogs (females: 5.5%; 85/1534, males: 6.2%; 113/1833; *p* = 0.27) tested separately (Table 3). Since age was identified as a factor influencing seroprevalence in both cats and dogs, we further examined the binomial model incorporating sex and age. Our analysis revealed similar findings, showing no significant difference in seropositivity rates between sexes (see supplementary Table S2).

**TABLE 3:** Seroprevalence of IgG SARS‐CoV‐2 antibodies in blood samples from cats and dogs by sex from October 2020 through June 2021.

Sex	Cats	Cats seroprevalence (95% CI)	Dogs	Dogs seroprevalence (95% CI)	Cats + Dogs	Cats + Dogs seroprevalence (95% CI)
Female	78/827	9.4% (7.5–11.6)	85/1534	5.5% (4.4–6.8)	163/2361	6.9% (5.9–8.0)
Male	99/1009	9.8% (8.0–11.8)	113/1833	6.2% (5.1–7.4)	212/2842	7.5% (6.5–8.5)
Total	177/1836	9.6% (8.3–11.1)	198/3367	5.9% (5.1–6.7)	375/5203	7.2% (6.5–7.9)
OR	1.04 (0.76–1.43)		1.12(0.84–1.50)		1.09 (0.88–1.34)	
*p*‐value	7.84E‐01		4.43E‐01		4.40E‐01	

### 3.5 Seroprevalence Over Time

**FIGURE 3:**
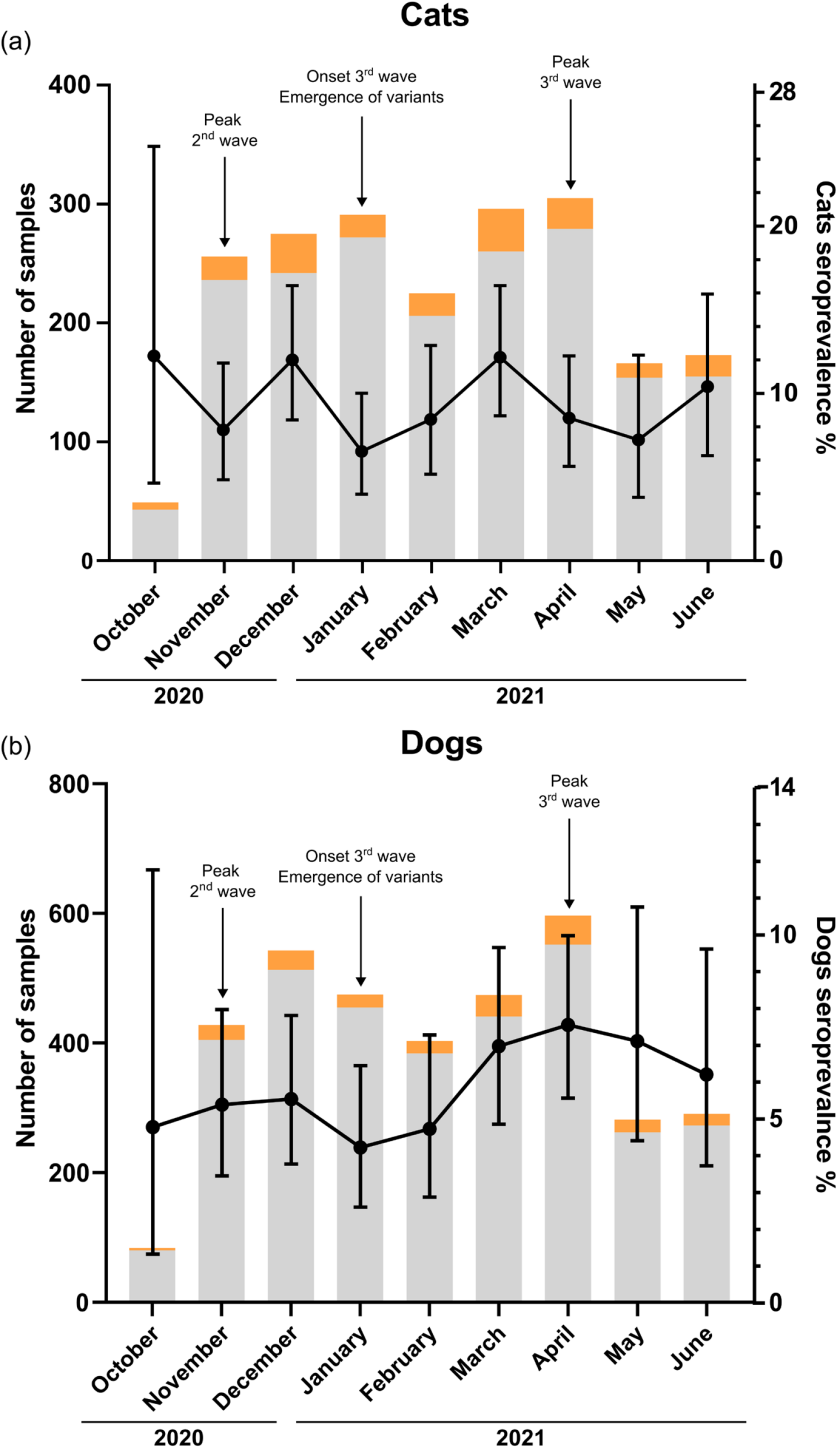
(a) Number of cat blood samples tested each month for anti‐SARS‐CoV‐2 antibodies by MIA from October 2020 through June 2021. Samples testing negative are shaded grey, and seropositive samples are in orange. Seroprevalence is represented by black dots, with 95% binomial CI (b) Number of dog blood samples tested each month for anti‐SARS‐CoV‐2 antibodies by MIA from October 2020 through June 2021. Samples testing negative are shaded grey, and seropositive samples are in orange. Seroprevalence is represented by black dots, with 95% binomial CI Notice that in this figure, dates have been pooled by calendar month for illustrative purposes, but in the statistical analysis, exact dates were used.

We next examined whether seroprevalence was associated with the time of sampling. For this analysis, we selected animals at least one year old at the date of sampling, resulting in a total number of 4174 pets (1530 cats and 2644 dogs) (Figure 3). Seroprevalence was not associated with the time of sampling for cats (OR = 1.52, 95% CI [0.56–1.15], *p*‐value = 0.41). However, seroprevalence among dogs increased over the 9 months of the study (OR = 3.47, 95% CI [1.47–8.23], *p*‐value = 0.0045).

## 4 Discussion

This study presents a comprehensive serological survey conducted on a large scale, focusing on pets (cats and dogs) to detect anti‐SARS‐CoV‐2 IgG antibodies. Sample collection took place in metropolitan France from October 2020 to June 2021. This timeframe notably spanned the peak of the second wave, the emergence of several variants (including Alpha and Beta) and marked the onset and peak of the third wave in France.

From a sample of 5613 pets, we report a seroprevalence of anti‐SARS‐CoV‐2 antibodies of 7.1%. We observed that only half of the samples (48%) were positive for both tri‐s and RBD, indicating that the RBD assay may be less sensitive than the tri‐s assay. This may be explained by the fact that the full trimeric spike antigen may bind a broader range of antibodies compared to the receptor‐binding domain, which includes only a small part of the spike protein. We found neutralising antibody activity in the sera of only 26% of seropositive pets. Cats were more likely to produce neutralising antibodies than dogs, which is likely associated with a more prolonged and intense immune stimulation in cats (Bosco‐Lauth et al. 2020). In humans, disease severity is positively correlated with neutralising antibody levels (Garcia‐Beltran et al. 2021).

In cats, we found a higher seroprevalence (9.3%) than previously observed in other European countries, which ranged from 0% to 6.4% (Barroso‐Arévalo et al. 2021a; Barroso‐Arévalo et al. 2021b; Michelitsch et al. 2020; Anna Michelitsch et al. 2021; Pomorska‐Mól et al. 2021; Schulz et al. 2021; Smith et al. 2021; Stevanovic et al. 2020; Zhao et al. 2021). However, most of these studies were done before the second wave, during a period of relatively lower viral circulation than our sampling period. In addition, most of these studies used a seroneutralisation assay.

In dogs, the observed seroprevalence (5.9%) is in accord with a previous study in France showing a prevalence of 4.8% in companion and military working dogs sampled between February 2020 and February 2021 (Laidoudi et al. 2021). Other studies looking for SARS‐CoV‐2 antibodies in dogs have reported seroprevalences ranging from 0% to 14.5% (Barroso‐Arévalo et al. 2021a; Barroso‐Arévalo et al. 2021b; Pomorska‐Mól et al. 2021; Smith et al. 2021; Stevanovic et al. 2021; Stevanovic et al. 2020; Zhao et al. 2021).

Importantly, we observed a significantly higher seroprevalence of anti‐SARS‐CoV‐2 antibodies in cats than in dogs (*p* = 4.2e‐08). Previous studies with fewer samples have found either no significant difference between species (Barroso‐Arévalo et al. 2021a; Calvet et al. 2021; Fritz et al. 2021a; Fritz et al. 2021b; Patterson et al. 2020; Pomorska‐Mól et al. 2021; Stevanovic et al. 2020) or that cats have significantly higher seroprevalence than dogs (Barroso et al. 2022; Colitti et al. 2021; Hamer et al. 2021).

Our study of a very large population of dogs and cats in natural conditions provides some evidence that cats are more exposed to SARS‐CoV‐2 infection than dogs, at least during the time frame of our sampling period. Potential causes of species differences in susceptibility between cats and dogs are numerous but likely include a variety of biological and behavioural factors, as well as differences in exposure. Interestingly, ACE‐2 shows greater sequence similarity between cat and human orthologues than observed between dogs and humans (Damas et al. 2020). The absence of data such as pet lifestyle (Indoor/Outdoor) or the frequency and nature of contacts with humans and other animals restricts our ability to identify a potential cause of the observed difference. In previous studies, most infected pets were epidemiologically linked to humans who had tested positive for COVID‐19 (Maurin et al. 2021).

In terms of age, we observed a higher seroprevalence among younger animals (between 0 and 3 years) for both species, which then decreased with age. A study of dogs sampled from the general population found seroprevalence was the highest in animals aged 5–6 years and that in COVID‐19+ households, seroprevalence peaked in slightly younger dogs, aged between 1 and 5 years (Stevanovic et al. 2021). Other studies have reported no significant associations with age in cats and dogs (Barroso et al. 2022; Pomorska‐Mól et al. 2021). An experimental study in cats found that juveniles appear more vulnerable than subadults (Shi et al. 2020). The decreasing seroprevalence we observed with age could also arise from age‐dependent behavioural changes. For example, young animals (< 3 years old) are more active and curious and may be in greater contact with their owners than older animals that prefer to remain quieter. The decrease could also reflect immunosenescence in older animals, as observed in humans (Bartleson et al. 2021).

We did not observe significant sex differences in seroprevalence in either species. Our findings are consistent with most previous studies also reporting an absence of sex differences in dogs and cats (Barroso et al. 2022; Calvet et al. 2021; Jairak et al. 2022; Pomorska‐Mól et al. 2021). A smaller study of 188 dogs and 61 cats found higher seropositivity in male dogs and an absence of a sex difference in cats (Patterson et al. 2020). Another study found that male dogs sampled from the general population were more likely to test positive than females, but this difference was not observed in dogs from COVID‐19+ households (Stevanovic et al. 2021). There is little evidence of a significant sex difference in susceptibility in humans. However, men are more likely to be affected by severe forms of COVID than women for a variety of reasons (Bechmann et al. 2022).

Interestingly, we observed a slight increase in seroprevalence in dogs during the study's nine months of sampling, a trend not observed among cats. Moreover, despite the occurrence of different events during our sampling period—such as the peaks and onset of two waves and the emergence of first variants—the exposure of cats and dogs does not seem to have been significantly impacted by these events. We expected an increase because antibodies have a longer persistence in the organism than viral RNA; thus, animals sampled at later dates would represent an accumulation of cases. The absence of a positive association between seroprevalence and the time of sampling in cats has been reported in two other studies in Europe, but conclusions were limited by the small number of samples collected over just a few months (Adler et al. 2022; Schulz et al. 2021). The absence of an association in cats suggests a limited persistence of antibodies in cats than in dogs. Few studies have investigated variation in the persistence of antibodies in animals. For example, a study carried out on seven dogs and two cats infected in natural conditions showed persistence of neutralising antibodies up to 10 months after infection in four of the dogs and the two cats, but also that persistence was markedly reduced in two of the dogs after three months (Decaro et al. 2021). Moreover, a study of two cats found that neutralising antibodies had disappeared by 110 days (Zhang et al. 2020). Based on these data, one possible reason for the lack of increase in seroprevalence during our study period could be a progressive seroreversion of infected cats that is equally compensated by the number of new infections, that is, seroconversion. If so, this would mean that the observed seroprevalence is not an accurate reflection of the total number of infections, at least in cats, during the whole epidemic. Instead, seroprevalence provides a snapshot of infections acquired during a time period. This also suggests that the seroprevalence observed in our study may underestimate the actual proportion of cats infected during the entirety of the epidemic. In order to enhance our comprehension of antibody persistence in cats and dogs, a longitudinal serological study involving a larger number of cats and dogs has to be conducted.

This study boasts numerous strengths, such as the nationwide recruitment of pets from various regions of France, a substantial sample size, the employment of multiple assays—including two serological methods—and meticulous control for several covariates. This study also has several limitations. Firstly, the inability to investigate the clinical history of animals due to variability in how each veterinarian documented this information poses a notable limitation. This variability may have introduced bias into our analysis by omitting consideration of potentially relevant clinical factors. Secondly, the absence of age and sex data for all pets restricted the number of animals analysed when these variables were studied. Third, the sample size from certain regions largely depended on the number of veterinarians collaborating with VEBIO in those areas. Small sample sizes may have resulted in insufficient statistical power to detect differences in seroprevalence between species, thus constraining the interpretation of regional results. Fourth, owing to our sample collection method, extrapolation of our findings to the SARS‐CoV‐2 infection history of pet owners is not feasible.

Human‐to‐pet transmission may promote viral adaptation, facilitating re‐infection with novel viral strains in humans (Bashor et al. 2021). Although suspected cases of infection from pet cats to humans have been reported, the substantial population of pet cats and their frequent close interactions with humans significantly increase the likelihood of such transmission events. While pets do not currently appear to be contributing to the SARS‐CoV‐2 pandemic, our results highlight the importance of SARS‐CoV‐2 exposure and possible subsequent infection in pets. Combined with the size of domestic cat and dog populations and the close contact with their human companions, our results highlight the importance of collecting more data on SARS‐CoV‐2 transmissibility and pathogenicity in companion animals. Also, when a SARS‐CoV‐2 infection is suspected in a pet, we still suggest collecting a sample for RT‐qPCR confirmation of infection, followed by whole‐genome sequencing to identify new mutations, particularly in antigenic sites targeted by the immune system. Similar public health recommendations applied to humans should also be implemented for animals to prevent human‐to‐animal transmission, such as not having contact with animals when a household member is COVID‐19 positive. Finally, as more individuals worldwide live in close proximity to companion animals, this study underscores the significance of the One Health concept, particularly in considering companion animals from the outset of an epidemic caused by a zoonotic disease.

## Author Contributions

M.F., P.B., S.G.R., A.B.‐M., V.L. and E.M.L. conceived and designed the study. M.F., E.E., D.d.R.d.F., D.G., S.D., B.B. and V.L designed and performed the experiments. All authors analysed the data and interpreted and discussed the results. M.F. and E.L. wrote the manuscript with input from all authors. All authors have read and agreed to the published version of the manuscript.

## Ethics Statement

According to the act of ‘use of live animals for scientific purposes’ effective in France on 14 January 2022, ethical approval was not sought or required since all pets were sampled by a veterinarian during a healthcare visit. All applicable international and national guidelines for the care of pets were followed.

## Conflicts of Interest

The authors declare no conflicts of interest.

## Supporting information


**Table S1.** Distribution of positive results in blood samples from cats and dogs by antigen from October 2020 through June 2021.

**Table S2.** Seroprevalence of IgG SARS‐CoV‐2 antibodies in blood samples from cats and dogs by sex from October 2020 through June 2021 using a binomial model incorporating sex and age.

## Data Availability

The data that support the findings of this study are openly available in zenodo at https://zenodo.org/records/8125085, reference number 10.5281/zenodo.7642801.
