# Supplementary material for: A Large‐Scale Serological Survey in Pets From October 2020 Through June 2021 in France Shows Significantly Higher Exposure to SARS‐CoV‐2 in Cats Compared to Dogs
Source: Zoonoses Public Health. 2024 Dec 8;72(2):184–93. doi: 10.1111/zph.13198 (PMC11772911; doi:10.1111/zph.13198)
Supplement: Supplementary file 1 — Table S1. Distribution of positive results in blood samples from cats and dogs by antigen from October 2020 through June 2021. [file ZPH-72-184-s001.docx]

**Supplementary table 1.** Distribution of positive results in blood samples from cats and dogs by antigen from October 2020 through June 2021.

|  | RBD | S tri | RBD and S tri | **Total** |
| --- | --- | --- | --- | --- |
| Cats | 23 | 111 | 55 | **189** |
| Dogs | 20 | 134 | 58 | **212** |
| **Total** | **43** | **245** | **113** | **401** |
