# Supplementary material for: A Large‐Scale Serological Survey in Pets From October 2020 Through June 2021 in France Shows Significantly Higher Exposure to SARS‐CoV‐2 in Cats Compared to Dogs
Source: Zoonoses Public Health. 2024 Dec 8;72(2):184–93. doi: 10.1111/zph.13198 (PMC11772911; doi:10.1111/zph.13198)
Supplement: Supplementary file 2 — Table S2. Seroprevalence of IgG SARS‐CoV‐2 antibodies in blood samples from cats and dogs by sex from October 2020 through June 2021 using a binomial model incorporating sex and age. [file ZPH-72-184-s002.docx]

Supplementary table 2. Seroprevalence of IgG SARS-CoV-2 antibodies in blood samples from cats and dogs by sex from October 2020 through June 2021 using binomial model incorporating sex and age.

| **Sex** | **Cats** | **Cat seroprevalence (95% c.i.)** | **Dogs** | **Dog seroprevalence (95% c.i.)** | **Cats + Dogs** | **Cats + Dogs seroprevalence (95% c.i.)** |
| --- | --- | --- | --- | --- | --- | --- |
| Female | 58/670 | 8.7% (6.6-11.0) | 64/1193 | 5.4% (4.2-6.8) | 122/1863 | 6.5% (5.5-7.8) |
| Male | 80/827 | 9.7% (7.7-11.9) | 94/1449 | 6.5% (5.3-7.9) | 174/2276 | 7.6% (6.6-8.8) |
| **Total** | **138/1497** | **9.2% (7.8-10.8)** | **158/2642** | **6.0% (5.1-7.0)** | **296/4139** | **7.2% (6.4-8.0)** |
| OR | 1.08 (0.75 - 1.54) | | 1.20 (0.87 - 1.67) | | 1.16 (0.91 - 1.47) | |
| P-value | 6.80E-01 | | 2.68E-01 | | 2.40E-01 | |
